# Electrical impedance myography in healthy dogs: Normative values, repeatability, and the impact of age

**DOI:** 10.3389/fvets.2022.1025528

**Published:** 2022-12-21

**Authors:** Sarah A. Verga, Sarbesh R. Pandeya, Joseph B. Kowal, Randall J. Cochran, Stefanie Lim, Julianna C. Sabol, Joan R. Coates, Seward B. Rutkove

**Affiliations:** ^1^Department of Neurology, Harvard Medical School, Beth Israel Deaconess Medical Center, Boston, MA, United States; ^2^Department of Veterinary Medicine and Surgery, College of Veterinary Medicine, University of Missouri, Columbia, MO, United States

**Keywords:** muscle, neuromuscular, canine, phase, atrophy

## Abstract

Convenient tools to assess canine skeletal muscle health would be useful for a variety of applications, including standard veterinary assessments of dog fitness, as well as studies of muscle deterioration due to age or disease. One technology that can be applied conveniently to awake dogs with minimal restraint is electrical impedance myography (EIM). In EIM, a weak electrical current is applied *via* surface electrodes to a muscle of interest and consequent impedance characteristics of the muscle are obtained, providing insight into muscle condition and composition. In this study, we assessed a total of 73 dogs (42 males and 31 females), of varied neutering status and breed, ages 0.6 to 13.5 years. We identified age-dependent reference values for the 100 kHz phase value in three pelvic limb muscles, caudal sartorius, cranial tibial, and gastrocnemius. While phase values were generally higher in males than females, the difference did not reach significance. In general, values declined on average with age at about 0.5 degrees/year, but with the decline being most substantial in the oldest dogs. Limited reproducibility assessment of the technique suggested good repeatability with variation in values between measurements being under 5%. These results show that EIM has the potential for the assessment of canine muscle health and may find value in aging muscle research.

## 1. Introduction

Loss of muscle mass can occur during many catabolic physiological and pathophysiological processes, including physical inactivity and starvation or anorexia, associated with systemic disease. Muscle loss associated with disease is called cachexia, whereas muscle loss associated with aging in the absence of disease is termed sarcopenia (1). Sarcopenia reflects loss of myofibers due to apoptosis and decreases in myofiber size. Overall muscle circumference is often maintained during early sarcopenia, as lost myofibers are replaced with adipose and fibrous tissue (1). Biochemical assessment of aging canine muscle has confirmed that the upregulation of autophagy occurs in aged dogs leading to myofiber loss (2). Muscle loss is common in animals with chronic diseases (e.g., chronic kidney disease, congestive heart failure, and cancer) or an acute injury or illness and during aging. Because older animals are more likely to develop chronic diseases, sarcopenia and cachexia can occur concurrently.

Visual scoring systems have been used as subjective measures to evaluate body and muscle condition in dogs. While many veterinarians use visual body condition scores (BCS) in which dogs' body condition ranges from 1 (very thin) to 5 (ideal) and to 9 (very overweight) to determine whether dogs are at a correct weight, these systems have significant limitations (3). An important limitation is the fact that visual scores do not adequately differentiate between increased body size due to fat vs. muscle. Animals can have significant muscle loss if overweight (BCS >5). Conversely, animals can have a low BCS (<4) but have minimal muscle loss. Muscle condition score (MCS) is 4-point scale graded as normal, or mild, moderate or severe loss (4). MCS had substantial repeatability and moderate reproducibility for assessment of muscle mass in dogs (5). Thus, body condition score, which is an assessment of fat, and MCS, which is an assessment of muscle, are not directly related.

Clinically relevant methods for quantifying muscle loss are needed. Ultrasonography and computed tomography (CT) of muscle has been used for assessment of epaxial muscles in healthy dogs. An ultrasonographic method for assessment of epaxial muscles has been validated for use on healthy dogs (6). Mean epaxial muscle area measured by ultrasonography was significantly lower in older dogs, compared with younger dogs, whereas epaxial muscle area measured by CT was only significantly lower in older dogs after normalization for vertebral height (7). In addition to loss of skeletal muscle mass, low muscle CT attenuation values suggested that older dogs also had greater muscle fat content than did younger dogs (8).

Electrical impedance myography (EIM) is a tool that has the potential to evaluate individual muscles non-invasively and painlessly, making it possible to use in awake dogs (9, 10). It relies on the application of a very weak, high frequency electrical current to a region of tissue and measurement of the resulting voltages, from which various electrical properties (the impedance) can be inferred. The general concept of EIM is that skeletal muscle can be modeled as a network of resistors and capacitors, with the intracellular and extracellular matrices of muscle tissue acting as resistors and the lipid bilayers that constitute the muscle membranes acting as capacitors. Thus, any global atrophy that reduces the cross-sectional area of muscle tissue would also be expected to increase the resistance (R); loss of both free extracellular and intracellular water will also contribute to increased resistance. As a muscle atrophies, the local capacitance of the muscle increases (more membranes per unit volume of muscle). The capacitance is inversely proportional to reactance (X), and myofiber atrophy, therefore, would be expected to decrease X. Phase angle (or simply phase) (θ) combines R and X into a single value by the equation θ = arctan (X/R) as is the most commonly used impedance parameters in EIM studies (11, 12). The advantage of the phase angle is that, to some extent, it corrects for simple volumetric alterations in the tissue, since simply decreasing the size of the muscle will have the effect of increasing X and R to a similar extent. The impedance (Z) itself, is calculated *via* the Pythagorean theorem (Z = sqrt (R^2^ + X^2^)); however, the impedance itself is rarely used in most applications since R is considerably larger than X and thus impedance tends simply to reflect R. It is a useful outcome parameter since it normalizes to some extent variations in muscle size and electrode geometry. We specifically provide the data on phase (and not resistance or reactance) because phase represents a more inherent muscle property than resistance or reactance values which are far more dependent on electrode size, inter-electrode distances, and muscle size and shape. The integrity of individual cell membranes, myofiber size, and the presence of fat and connective tissue has a significant effect on the tissue's impedance; consequently, a muscle's impedance can be used to measure the tissue's alteration in disease. Indeed, impedance has been shown to change in a variety of neuromuscular conditions and can be used as an overall marker of muscle health (11, 12).

Comparative aging research in companion animals that share the same environment, diet, and exposure to pollutants, etc., as humans may make the canine a more realistic “real-world” model than laboratory rodents. The objective of this study was to assess normal EIM values in selected pelvic limb muscles of healthy dogs, determine its association with age, including preliminarily developing a range of age associated reference values, and assess sex differences and reproducibility.

## 2. Materials and methods

### 2.1. Study population

Healthy dogs were recruited by two different mechanisms. One group was recruited at the University of Missouri Veterinary Health Center. These dogs were included in the study if they had no history of neurologic or orthopedic disease, a normal complete blood count and serum biochemistry, and a normal neurologic examination. In addition, boxers were recruited at the American Boxer National Specialty. These dogs were included if they had normal orthopedic and neurologic examinations. All studies were completed with Institutional Animal Care and Use Committee approval and informed consent from the owners. Only dogs with a BCS of 4, 5 and 6 (on the standard 1-9 scale) were included in the data analysis to reduce the potential impact of excessive body fat on the measurements. Both groups of dogs were studied at single session for practical reasons, as it is very difficult to get these pet dogs back for repeated visits over a short period of time.

### 2.2. Electrical impedance myography acquisition

**Figure 1:**
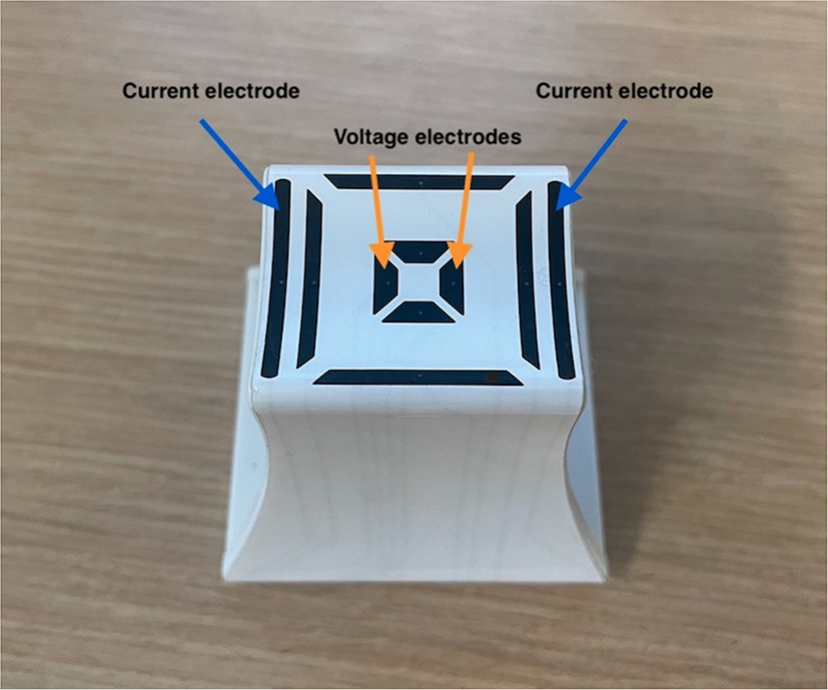
Electrode array used to obtain data. The outer electrodes (blue arrows) emit the electrical current and the inner electrodes (orange arrows) measure the resulting voltages.

A handheld EIM device (mView, Myolex, Inc, Boston, MA) was utilized to obtain impedance measurements from the dogs' hind limb muscles using a small electrode array originally designed for human infants (13). This electrode has three electrode sets embedded within it (Figure 1), two providing current flow along the myofibers and one across myofibers. Based on standard impedance theory (12), the farther apart the electrodes, the great depth of muscle penetration. Thus, only the set that was spaced farthest apart was utilized in this analysis (the outer electrodes for longitudinal measurements, see Figure 1). Five pelvic limb muscles were initially chosen for study based on their easy palpability, superficial location, and size. These included, gastrocnemius, cranial tibial, gracilis, caudal sartorious, and biceps femoris; however, given limitations on testing, including inconsistent values and more limited data sets in gracilis and biceps femoris, results on only three are reported here: cranial tibial, caudal sartorious and gastrocnemius. During data collection, the dogs were awake and gently restrained in lateral recumbency. Clippers were used to remove the hair over the targeted muscle, and saline was used to moisten the skin allowing for effective electrical current conduction from the EIM probe. The probe was placed at the thickest part of the targeted muscle. The skin was re-moistened with a saline-soaked sponge in between each measurement. Three trials of repeated recordings were performed to test the measurements' repeatability. The left side was studied uniformly and the right side less consistently across all dogs. Thus, in this analysis, only left side muscle data is provided.

### 2.3. EIM data processing

We extracted the 100 kHz values from the multifrequency data set. The third measurement served as the final data set analyzed and the first, second and third were used in the intrarater reproducibility assessments. The main target EIM parameter i.e., phase value was our primary measure for data inclusion and exclusion criteria as any phase value that were negative or >30° were excluded as spurious. Similarly, any values that were two standard deviations away from the mean for that muscle were also excluded. Overall, 15 out of 219 (i.e., 6.89%) EIM muscle measurements were filtered and removed. Single muscle data is analyzed as well as the average of 100 kHz data from all three muscles in the limb.

### 2.4. Statistical analysis

Analysis was conducted in R 4.2.1 (R Foundation for Statistical Computing, Vienna, Austria). Given its relative independence from simple volumetric effects, we chose the phase value as the primary outcome measure. For this analysis, we used second-degree polynomial regression to observe the quadratic trend of phase value through the fitted regression line along with its lower prediction limit, based on the 95% confidence interval; this regression provided an age-specific lower limit of normal values for each muscle specific muscle. In addition, after assessing for normality with the Kolmogorov-Smirnov test and QQ plots, we used the Wilcoxon rank sum test to conduct comparison tests between the two sexes (male vs. females). To assess the reliability of the EIM measures, we also performed the coefficient of variation (CoV) assessments on the first, second and third repeated measurements from each muscle and their average.

## 3. Results

### 3.1. Study population

A total of seventy-three dogs were included in the study, with forty-two males and thirty-one females, all with body conditioning scores between 4 and 6. Of the 42 males, 42 gastrocnemius, 42 caudal sartorius and 20 cranial tibial muscle measurements were obtained. Of the 31 females, 31 gastrocnemius, 30 caudal sartorius and 17 cranial tibial muscle measurements were obtained. Not all muscles were able to be measured due to various circumstances including time restraints, dog owner consent, fur shaving limitations for the show dogs, and other variables. There were different breeds of dogs included in the analysis. These were: Australian Cattle Dog (*n* = 1), Australian Shepherd (*n* = 1), Beagle (*n* = 1), Boxer (*n* = 59), Chinese Crested-Beagle mix (*n* = 1), German Shepherd Dog (*n* = 2), Great Dane (*n* = 1), Greyhound (*n* = 1), Husky mix (*n* = 1), Jack Russel Terrier (*n* = 1), Labrador Retriever mix (*n* = 1), mixed-breed (*n* = 1), Pit Bull mix (*n* = 1), Rat Terrier (*n* = 1) and Treeing Walker Coonhound (*n* = 1). All the Boxers were show dogs and their data was collected on site at the American Boxer National Specialty. However, the remaining dogs were pet volunteers and underwent data collection at the University of Missouri Veterinary Health Center during routine clinical visits. As described in detail in Table 1, mean age was 4.3 years, mean weight of 28.7 kgs, and mean BCS of 4.9. Table 1 further summarizes the number of neutered dogs along with means of age, weight and BCS; neutered dogs tended to be younger than intact animals.

**Table 1:** Mean and standard deviation for age, weight, and BCS for both neutered and intact dogs.

	**Females**			**Males**			**Total**
	**Intact females**	**Neutered females**	**Total**	**Intact males**	**Neutered males**	**Total**	**Total**
	**(*N* = 20)**	**(*N* = 11)**	**(*N* = 31)**	**(*N* = 31)**	**(*N* = 11)**	**(*N* = 42)**	**(*N* = 73)**
Age (years) mean (±SD, range)	2.8 (±2.08, 0.63–7.72)	8.2 (±4.45, 1.05–13.6)	4.3 (±3.58, 0.62–13.5)	3.2 (±3.20, 0.76–11.2)	7.3 (±3.95, 2.0–13.0)	3.9 (±3.56, 0.76–13.0)	4.32 (±3.5, 1–13)
Weight (kg) mean (±SD, range)	26.1 (±3.6, 20.4–31.87)	26.8 (±10.9, 7.7–54.1)	26.8 (±3.2, 7.7–54.1)	31.0 (±3.7, 24.1-41.4)	20.3 (±10.6, 6.8–33.6)	30.3 (±4.2, 6.8–41.4)	28.7 (±1.6, 6.8–54.1)
BCS mean (±SD, range)	4.7 (±0.57, 4–6)	5.2 (±0.75, 4–6)	5.0 5.0 (±0.52, 4–6)	4.8 (±0.45, 4–6)	5.5 (±0.52, 5–6)	4.9 4.9 (±0.4, 4–6)	4.9 (±0.4, 4–6)

### 3.2. Evaluation of EIM phase with age in male and female dogs: Establishing lower limits of normal

**Figure 2:**
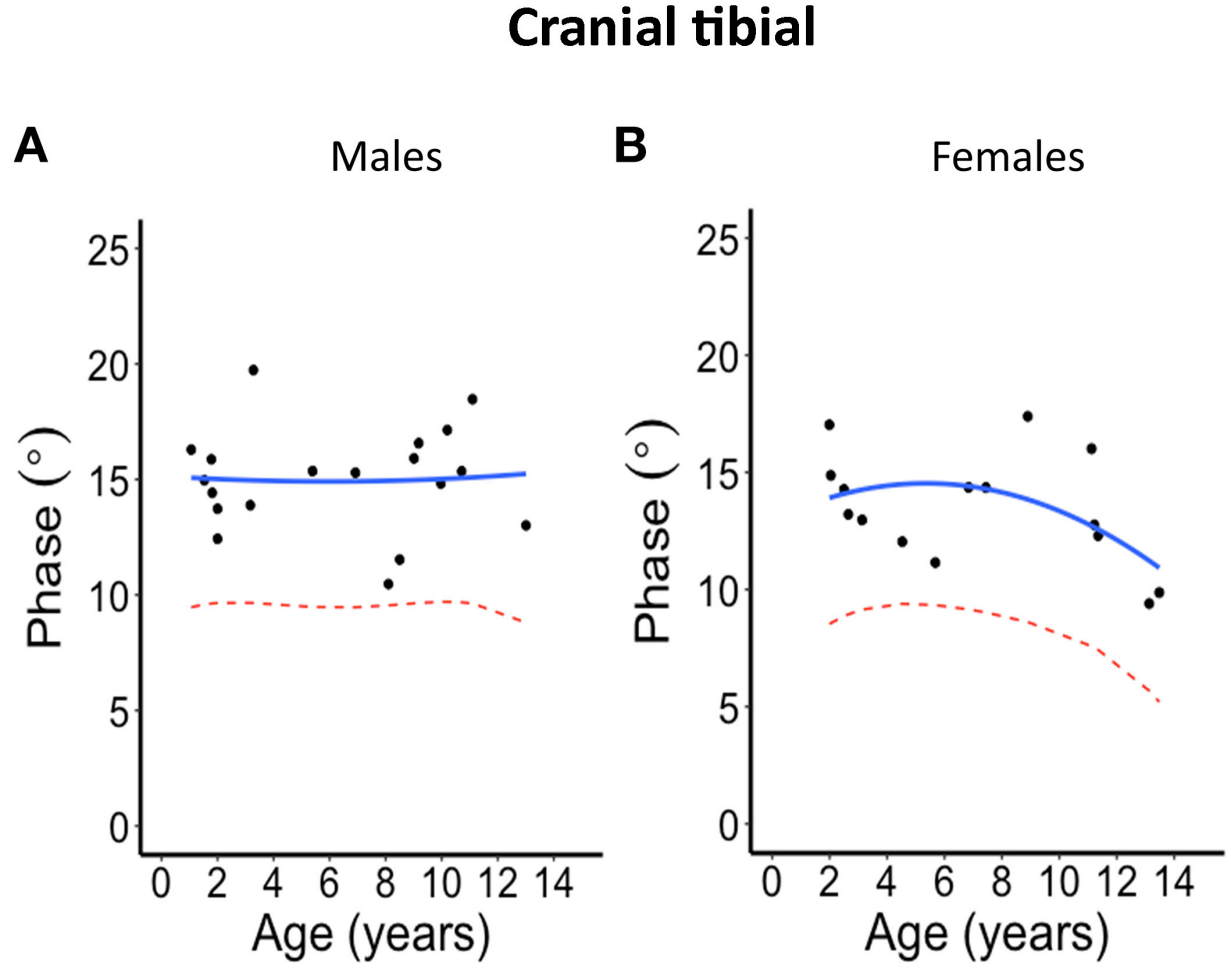
Phase angle values at 100 kHz made on cranial tibial muscle for **(A)** males and **(B)** females. The blue solid line represents the normal fit generated from the second-degree polynomial regression through the log-transformed data while the red dotted lines represent the parallel curve that is the lower limit of normal based on the 95% prediction limit of the quadrative curve.

**Figure 3:**
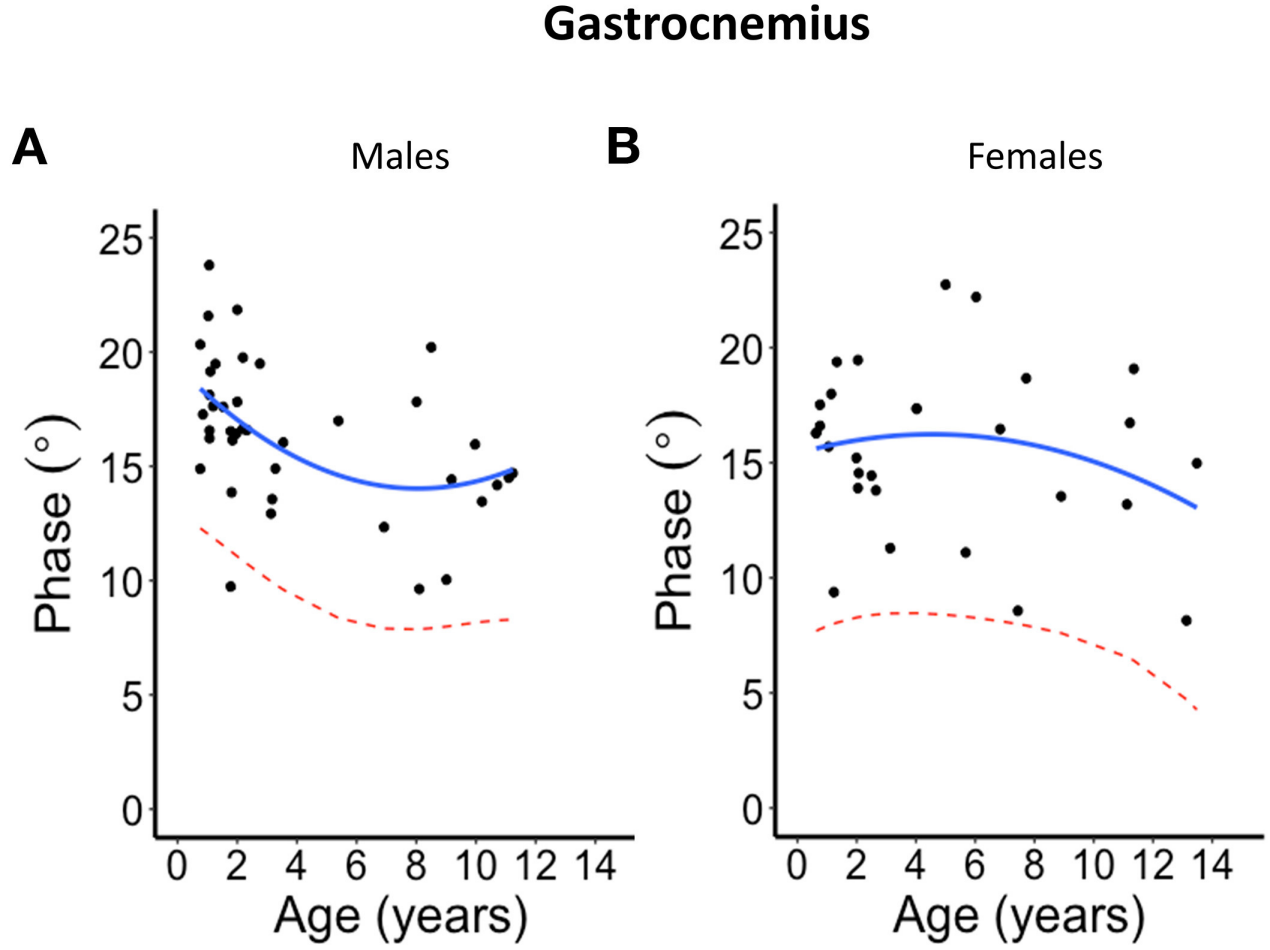
Phase angle values at 100 kHz made on gastrocnemius muscle for **(A)** males and **(B)** females. The blue solid line represents the normal fit generated from the second-degree polynomial regression through the log-transformed data while the red dotted lines represent the parallel curve that is the lower limit of normal based on the 95% prediction limit of the quadrative curve.

**Figure 4:**
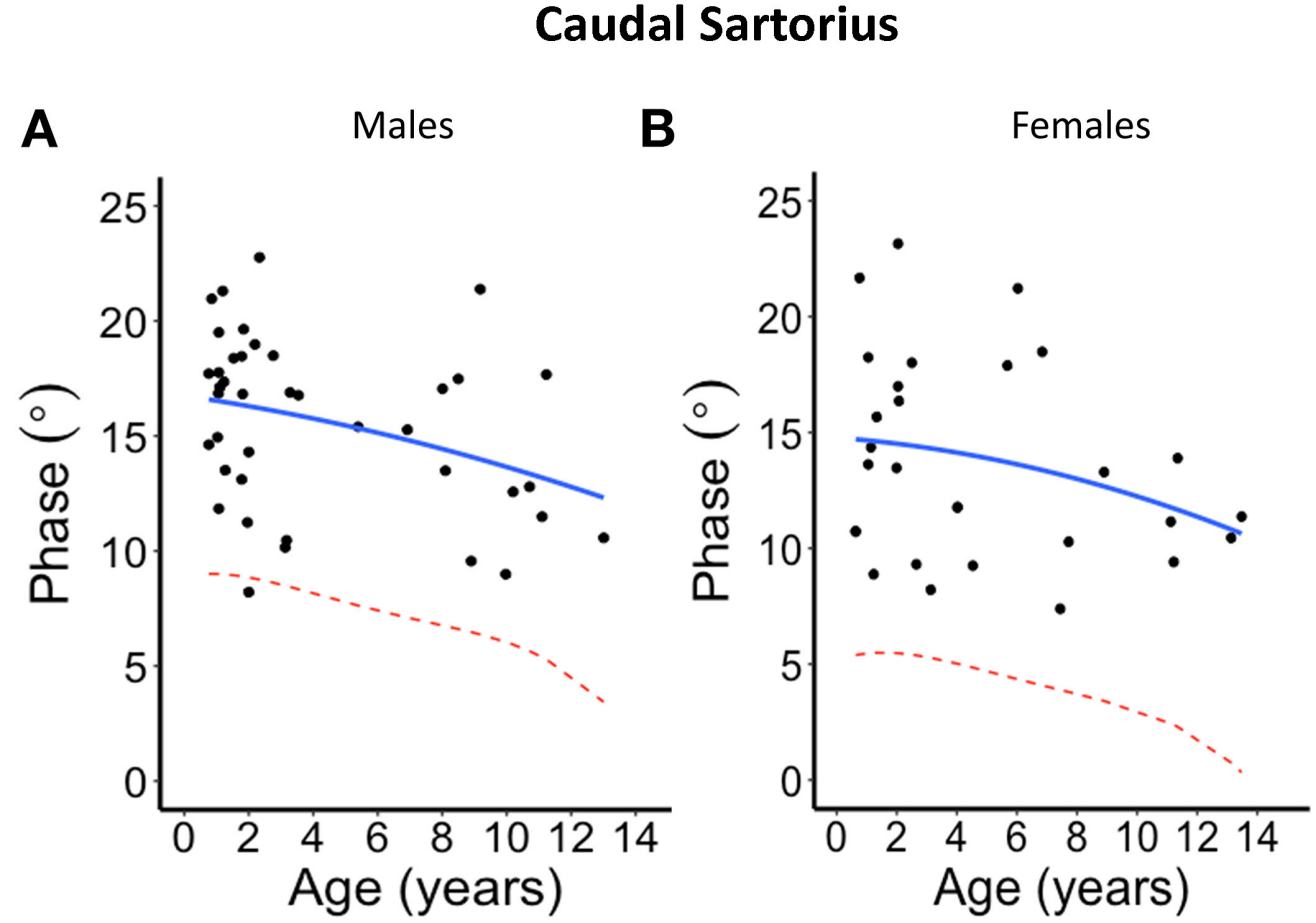
Phase angle values at 100 kHz made on caudal sartorius muscle for **(A)** males and **(B)** females. The blue solid line represents the normal fit generated from the second-degree polynomial regression through the log-transformed data while the red dotted lines represent the parallel curve that is the lower limit of normal based on the 95% prediction limit of the quadrative curve.

**Figure 5:**
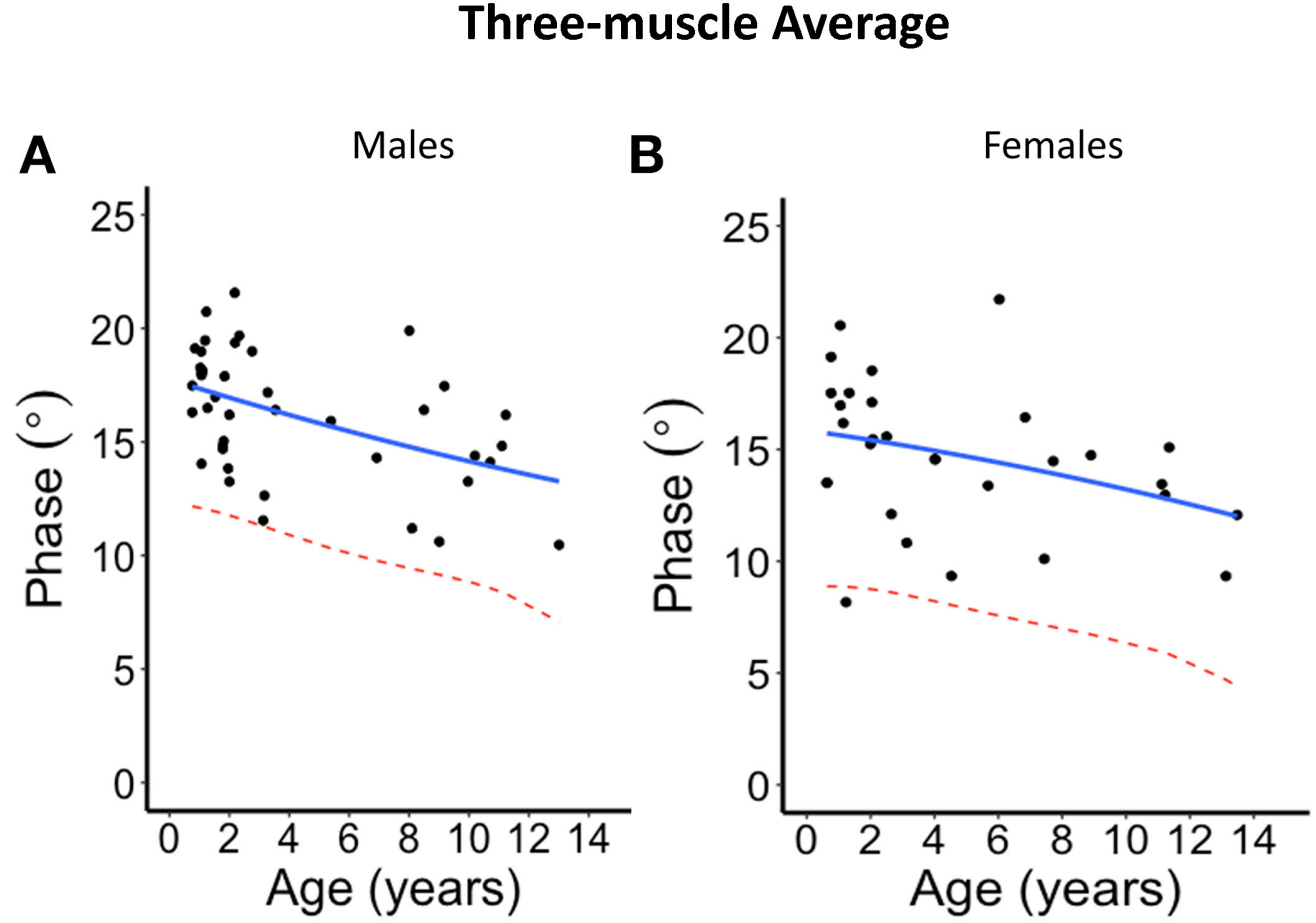
Phase angle values at 100 kHz made on average of the three muscles for **(A)** males and **(B)** females. The blue solid line represents the normal fit generated from the second-degree polynomial regression through the log-transformed data while the red dotted lines represent the parallel curve that is the lower limit of normal based on the 95% prediction limit of the quadrative curve.

The second-degree polynomial regression result shows the normal linear or parabolic pattern, and its lower limit fit as given in Figures 2–5 for each muscle and their average for different sexes, as well the lower 95% confidence limit, that can be defined as the lower limit of normal. Despite the relative small sample size, it was evident from all these graphs that phase tends to decrease with age. This is further summarized in Table 2 and provided with age-specific lower limits of normal.

**Table 2:** Lower limit of 95% prediction from the second-degree polynomial regression showing phase reduction with increasing age.

**Muscle**	**Sex**	**Age (years)**						
		**1**	**3**	**5**	**7**	**9**	**11**	**13**
Gastrocnemius	Female	7.9°	8.4°	8.4°	8.1°	7.6°	6.6°	4.9°
	Male	12.1°	10.1°	8.6°	7.9°	8.0°	8.3°	8.0°
Cranial tibial	Female	7.6°	9.1°	9.4°	9.1°	8.6°	7.7°	5.9°
	Male	9.4°	9.7°	9.5°	9.5°	9.6°	9.6°	8.8°
Caudal sartorius	Female	5.5°	5.3°	4.7°	4.9°	3.4°	2.5°	0.9°
	Male	9.0°	8.5°	7.8°	7.1°	6.4°	5.5°	3.5°
Average of 3 muscles	Female	8.9°	8.5°	7.9°	7.3°	6.7°	6.0°	4.8°
	Male	12.1°	11.3°	10.5°	9.7°	9.2°	8.4°	7.1°

### 3.3. Relationship of all EIM parameters to sex and age: A multivariate analysis

In the previous analysis, we generated a simple table (Table 2) by which one can identify normal vs. abnormal values for EIM separately by sex for the major EIM parameter of phase. Here we take another view at the data set by incorporating age and sex values into a multivariate regression. Table 3 summarizes these results for all three impedance parameters. As can be seen, of all three impedance parameters, resistance appears to be most age-sensitive, increasing significantly with age for all individual muscles and on average across all the muscles. Phase showed reductions in gastrocnemius and on average across all three muscles with age but not in the other muscles separately. Reactance showed no significant relationship with age. Although samples are relatively small, male and female animals did not show significant differences in this analysis.

**Table 3:** EIM parameter relationship to age and sex *via* multivariate regression, revealing significant relationships with age for some parameters, but not for sex.

**Muscle**	**EIM measure**	**Parameter**	**Estimate**	**Standard error**	***P*-value**	**Adjusted *R*-squared**
Gastrocnemius (*N* = 73)	Phase	Age	−0.347	0.104	0.001	0.125
		Sex	0.558	0.823	0.499	
	Resistance	Age	1.191	0.339	<0.001	0.149
		Sex	−2.888	2.326	0.218	
	Reactance	Age	0.024	0.090	0.78	0.017
		Sex	−0.684	0.666	0.308	
Cranialtibialis (*N* = 37)	Phase	Age	−0.127	0.108	0.251	0.03114
		Sex	1.036	0.859	0.237	
	Resistance	Age	0.829	0.235	0.001	0.252
		Sex	−2.2075	1.805	0.230	
	Reactance	Age	0.116	0.081	0.162	0.0018
		Sex	0.123	0.643	0.849	
Sartorius (*N* = 72)	Phase	Age	−0.1269	0.108	0.251	0.031
		Sex	1.0359	0.859	0.237	
	Resistance	Age	0.829	0.235	0.001	0.252
		Sex	−2.207	1.805	0.230	
	Reactance	Age	0.116	0.081	0.162	0.0018
		Sex	0.123	0.643	0.849	
Average (*N* = 73)	Phase	Age	−0.346	0.091	<0.001	0.170
		Sex	0.918	0.710	0.200	
	Resistance	Age	1.045	0.265	<0.001	0.194
		Sex	−3.027	1.815	0.100	
	Reactance	Age	0.076	0.063	0.24	0.026
		Sex	−0.741	0.495	0.14	

### 3.4. Within-session, single evaluator repeatability of EIM measurements (phase, resistance, and reactance)

To test the repeatability each of the EIM parameters, we used the measurements in the first, second and third trials in rapid succession, as described above, obtained by a single evaluator. Based on the assessment of the three muscle groups *via* coefficient of variation and mean percent difference, it was evident that EIM displayed good repeatability. A detailed summary of these values is given in Table 4.

**Table 4:** Single evaluator, within-session EIM repeatability assessments.

**Muscle**	**Value**	**Phase**	**Resistance**	**Reactance**
Gastrocnemius	Mean percent difference (%)	1.3	1.9	3.8
	Coefficient of variation	0.095	0.0342	0.091
Cranial tibial	Mean percent difference (%)	2.6	1.7	1.2
	Coefficient of variation	0.079	0.035	0.089
Caudal Sartorius	Mean percent difference (%)	2.1	0.98	1.9
	Coefficient of variation	0.090	0.049	0.099

## 4. Discussion

In this study, we were able to demonstrate a trend toward decreasing phase values with age in most pelvic limb muscles studied in both male and female dogs of varying breed. This reduction in values mirrors that observed in other animals, including mice (14) and humans (15, 16), where the technique has also been applied. Preliminary studies also show a similar trend in zebrafish (Rutkove, unpublished communication). The age-associated decline in EIM parameters, most generally apparent at the oldest ages, is consistent with, but not diagnostic of, sarcopenia. Of course, as we noted earlier, concomitant changes, including cachexia, possibly related to concurrent conditions such as undiagnosed neoplasm or cardiac dysfunction could also play a role. The relatively minor alterations in the cranial tibial in males with age is unclear.

Through our fitted models, we were able to also establish a lower limit of normal for these pelvic limb muscles, as we had done previously in humans and have also provided age-specific lower limits of normal based on the modeled 95% confidence limits (17). The data here could assist in identifying dogs with abnormalities in muscle if the values fall below the lower range of values. Of note, we do not provide an upper limit of normal, since nearly all muscle disorders would be expected to reduce the phase values, not increase them, with the exception, perhaps of myostatin mutations causing marked muscle hypertrophy as described in Whippets (18, 19).

We performed additional multivariate regression to assess the impact of sex and together on all the major impedance parameters. This analysis further supported the polynomial regression completed separately for the sexes described above but also showed that many of the age effects were mostly related to changes in resistance [which secondarily impacts phase, since phase = arctan (reactance/resistance)] and not reactance. It also suggested that there was no significant impact of sex in this analysis. Finally, our repeatability assessments, although limited, show acceptable values for all three impedance parameters.

This study had several limitations. First, to some extent these dogs represented a convenience sample of mixed breeds, sexes, and intact or neutered status. There were many Boxers as compared to other breeds because part of data collection was mainly limited to performance dogs present at a dog show. Also, we lacked multiple examiners and day-to-day measurement comparisons for all dogs and thus our repeatability assessments are fairly limited. Clearly, further studies will be needed to fully understand the real-world reproducibility of this technique. Second, while we overall have an acceptable number of animals for such an analysis (seventy-three), as one breaks it down by sex, clearly the numbers are not as large as might be desirable. Thus, the normative lower limits here provided, could only serve as a starting point for an eventually larger database. In addition, it is also possible that a contribution of increasing subcutaneous fat in the oldest dogs could play a role as well. We sought to exclude this possibility by including only dogs with healthy body conditioning scores of 4, 5, or 6, thus any confounding effect from this should have been limited. Nevertheless, it is impossible to exclude a contribution of subcutaneous fat to these results. Furthermore, we only looked at small set of pelvic limb (appendicular) muscles, and did not include any epaxial muscles typically assessed as part of screening of dog health, though there is no reason a similar approach could not be used. In addition, we only report normative values on a single impedance measure at a single frequency; clearly, similar analyses could have been performed separately for the resistance and reactance and at other frequencies. Next, the normative values established here are dependent on the specifics of the electrode array applied, including interelectrode distance and electrode size. Fortunately, the phase values tend to be less effected than other impedance parameters by these variations. In addition, we studied dogs no older than age 13.5 years. While this represents a typical life expectancy for many breeds, clearly extending these assessments to still older dogs would be interesting. Finally, we did not separate out sexually intact from neutered animals in our analyses since this would have further reduced our sample sizes in each category to such a point that the analyses would have lost considerable power.

There are several important applications to this initial work of applying EIM technology to the assessment of dog muscle health. First, it helps demonstrate that EIM has the potential serve as a useful tool to assess canine muscle condition across the ages and can be applied successfully to a number of dogs of various breeds. This could have value in the pet food industry as well as serving as an office tool by veterinarians to assess condition of show dogs or dogs with various medical ailments. Second, it could help in the assessment of dog models of human muscle disease, including dystrophin deficiency (as has already been studied with EIM to some extent (10)), myotubular myopathy (20), and nemaline myopathy (21). Finally, and perhaps most interesting, it could serve as useful tool for studies of aging more broadly, with specific application to human aging. Indeed, it is already currently being applied for this purpose as part of the Dog Aging Project (22).

## Data availability statement

The original contributions presented in the study are included in the article/supplementary material, further inquiries can be directed to the corresponding author.

## Ethics statement

The animal study was reviewed and approved by University of Missouri Institutional Animal Care and Use Committee; Protocol # 10077. Written informed consent was obtained from the owners for the participation of their animals in this study.

## Author contributions

JK and JC contributed to the conception and design of the study. JK, JC, RC, JS, and SL contributed to data collection. SR, SV, and SP performed the data analysis. SR wrote the first draft of the manuscript. All authors contributed to manuscript revision, read, and approved the submitted version.
